# Supplementary material for: Comparison of Eight Commercially Available Faecal Point-of-Care Tests for Detection of Canine Parvovirus Antigen
Source: Viruses. 2021 Oct 15;13(10):2080. doi: 10.3390/v13102080 (PMC8540396; doi:10.3390/v13102080)
Supplement: Supplementary file 1 [file viruses-13-02080-s001.zip › viruses-1383983-supplementary.pdf]

Supplementary material

Table S1. Age and vaccination status of the 150 dogs, results of eight point-of-care tests, real-time polymerase chain reaction (incl. virus load/g faeces) and virus culture for detection of canine parvovirus of 150 faecal samples (three groups; group H: healthy dogs, group S: shelter dogs, group p: dogs with suspicion of parvovirosis).

#	Age (in years)	Vac. status	Group	Snap® Parvo (POCT-A)	Fassisi® Parvo (POCT-B)	Primagnost® Parvo H + K (POCT-C)	FASTest® PARVO Card (POCT-D)	Vetexpert Rapid Test CPV Ag® (POCT-E)	Anigen Rapid CPV Ag Test Kit® (POCT-F)	ImmunoRun® Parvovirus Antigen Detection Kit (POCT-G)	WITNESS® Parvo (POCT-H)	qPCR	Virus load/g faeces	Virus culture
1	12	not last 4 weeks	S	-	-	-	-	-	-	-	-	+	2,15E+06	-
2	8	not last 4 weeks	S	-	-	-	-	-	-	-	-	-	-	-
3	9	not last 4 weeks	S	-	-	-	-	-	-	-	-	+	1,19E+06	-
4	9	not last 4 weeks	S	-	-	-	-	-	-	-	-	-	-	-
5	12	not last 4 weeks	S	-	-	-	-	-	-	-	-	-	-	-
6	12	not last 4 weeks	S	-	-	-	-	-	-	-	-	+	2,17E+06	-
7	4	not last 4 weeks	S	-	-	-	-	-	-	-	-	-	-	-
8	3	not last 4 weeks	S	-	-	-	-	-	-	-	-	-	-	-
9	9	not last 4 weeks	S	-	-	-	-	-	-	-	-	-	-	-
10	5	not last 4 weeks	S	-	-	-	-	-	-	-	-	-	-	-
11	7	not last 4 weeks	S	-	-	-	-	-	-	-	-	-	-	-
12	10	not last 4 weeks	S	-	-	-	-	-	-	-	-	+	4,65E+06	-

#	Age (in years)	Vac. status	Group	Snap® Parvo (POCT-A)	Fassisi® Parvo (POCT-B)	Primagnost® Parvo H + K (POCT-C)	FASTest® PARVO Card (POCT-D)	Vetexpert Rapid Test CPV Ag® (POCT-E)	Anigen Rapid CPV Ag Test Kit® (POCT-F)	ImmunoRun® Parvovirus Antigen Detection Kit (POCT-G)	WITNESS® Parvo (POCT-H)	qPCR	Virus load/g faeces	Virus culture
13	4	not last 4 weeks	S	-	-	-	-	-	-	-	-	-	-	-
14	4	not last 4 weeks	S	-	-	-	-	-	-	-	-	-	-	-
15	9	not last 4 weeks	S	-	-	-	-	-	-	-	-	-	-	-
16	4	not last 4 weeks	S	-	-	-	-	-	-	-	-	-	-	-
17	5	not last 4 weeks	S	-	-	-	-	-	-	-	-	-	-	-
18	11	not last 4 weeks	S	-	-	-	-	-	-	-	-	-	-	-
19	0.9	not last 4 weeks	S	-	-	-	-	-	-	-	-	-	-	-
20	6	not last 4 weeks	S	-	-	-	-	-	-	-	-	-	-	-
21	10	not last 4 weeks	S	-	-	-	-	-	-	-	-	-	-	-
22	4	not last 4 weeks	S	-	-	-	-	-	-	-	-	-	-	-
23	0.8	not last 4 weeks	S	-	-	-	-	-	-	-	-	-	-	-

24	12	not last 4 weeks	S	-	-	-	-	-	-	-	-	-	-	-
25	7	not last 4 weeks	S	-	-	-	-	-	-	-	-	-	-	-
26	11	not last 4 weeks	S	-	-	-	-	-	-	-	-	-	-	-
**#**	**Age (in years)**	**Vac. Status**	**Group**	**Snap® Parvo (POCT-A)**	**Fassisi® Parvo (POCT-B)**	**Primagnost® Parvo H + K (POCT-C)**	**FASTest® PARVO Card (POCT-D)**	**Vetexpert Rapid Test CPV Ag® (POCT-E)**	**Anigen Rapid CPV Ag Test Kit® (POCT-F)**	**ImmunoRun® Parvovirus Antigen Detection Kit (POCT-G)**	**WITNESS® Parvo (POCT-H)**	**qPCR**	**Virus load/g faeces**	**Virus culture**
27	2	not last 4 weeks	S	-	-	-	-	-	-	-	-	-	-	-
28	9	not last 4 weeks	S	-	-	-	-	-	-	-	-	-	-	-
29	1	not last 4 weeks	S	-	-	-	-	-	-	-	-	-	-	-
30	5	not last 4 weeks	S	-	-	-	-	-	-	-	-	-	-	-
31	2	not last 4 weeks	S	-	-	-	-	-	-	-	-	-	-	-
32	4	not last 4 weeks	S	-	-	-	-	-	-	-	-	-	-	-
33	9	not last 4 weeks	S	-	-	-	-	-	-	-	-	-	-	-
34	1	not last 4 weeks	S	-	-	-	-	-	-	-	-	+	2,51E+06	-
35	0.6	not last 4 weeks	S	-	-	-	-	-	-	-	-	+	5,48E+06	-
36	2	not last 4 weeks	S	-	-	-	-	-	-	-	-	-	-	-
37	2	not last 4 weeks	S	-	-	-	-	-	-	-	-	-	-	-
38	3	not last 4 weeks	S	-	-	-	-	-	-	-	-	+	4,83E+06	-
39	11	not last 4 weeks	S	-	-	-	-	-	-	-	-	-	-	-
40	10	not last 4 weeks	S	-	-	-	-	-	-	-	-	-	-	-
**#**	**Age (in years)**	**Vac. status**	**Group**	**Snap® Parvo (POCT-A)**	**Fassisi® Parvo (POCT-B)**	**Primagnost® Parvo H + K (POCT-C)**	**FASTest® PARVO Card (POCT-D)**	**Vetexpert Rapid Test CPV Ag® (POCT-E)**	**Anigen Rapid CPV Ag Test Kit® (POCT-F)**	**ImmunoRun® Parvovirus Antigen Detection Kit (POCT-G)**	**WITNESS® Parvo (POCT-H)**	**qPCR**	**Virus load/g faeces**	**Virus culture**
41	2	not last 4 weeks	S	-	-	-	-	-	-	-	-	-	-	-
42	11	not last 4 weeks	S	-	-	-	-	-	-	-	-	-	-	-
43	4	not last 4 weeks	S	-	-	-	-	-	-	-	-	-	-	-
44	12	not last 4 weeks	S	-	-	-	-	-	-	-	-	-	-	-
45	0.8	not last 4 weeks	S	-	-	-	-	-	-	-	-	-	-	-
46	8	not last 4 weeks	S	-	-	-	-	-	-	-	-	+	9,35E+05	-
47	5	not last 4 weeks	S	-	-	-	-	-	-	-	-	-	-	-
48	8	not last 4 weeks	S	-	-	-	-	-	-	-	-	+	5,27E+05	-
49	10	not last 4 weeks	S	-	-	-	-	-	-	-	-	-	-	-
50	9	not last 4 weeks	S	-	-	-	-	-	-	-	-	-	-	-

51	4	not last 4 weeks	H	-	-	-	-	-	-	-	-	-	-	-
52	11	not last 4 weeks	H	-	-	-	-	-	-	-	-	-	-	-
53	2	not last 4 weeks	H	-	-	-	-	-	-	-	-	+	1,20E+06	-
54	2	not last 4 weeks	H	-	-	-	-	-	-	-	-	-	-	-
**#**	**Age (in years)**	**Vac. status**	**Group**	**Snap® Parvo (POCT-A)**	**Fassisi® Parvo (POCT-B)**	**Primagnost® Parvo H + K (POCT-C)**	**FASTest® PARVO Card (POCT-D)**	**Vetexpert Rapid Test CPV Ag® (POCT-E)**	**Anigen Rapid CPV Ag Test Kit® (POCT-F)**	**ImmunoRun® Parvovirus Antigen Detection Kit (POCT-G)**	**WITNESS® Parvo (POCT-H)**	**qPCR**	**Virus load/g faeces**	**Virus culture**
55	4	not last 4 weeks	H	-	-	-	-	-	-	-	-	-	-	-
56	5	not last 4 weeks	H	-	-	-	-	-	-	-	-	-	-	-
57	3	not last 4 weeks	H	-	-	-	-	-	-	-	-	-	-	-
58	6	not last 4 weeks	H	-	-	-	-	-	-	-	-	+	7,05E+06	-
59	8	not last 4 weeks	H	-	-	-	-	-	-	-	-	-	-	-
60	3	not last 4 weeks	H	-	-	-	-	-	-	-	-	-	-	-
61	2	not last 4 weeks	H	-	-	-	-	-	-	-	-	-	-	-
62	10	not last 4 weeks	H	-	-	-	-	-	-	-	-	-	-	-
63	8	not last 4 weeks	H	-	-	-	-	-	-	-	-	-	-	-
64	6	not last 4 weeks	H	-	-	-	-	-	-	-	-	-	-	-
65	4	not last 4 weeks	H	-	-	-	-	-	-	-	-	-	-	-
66	2	not last 4 weeks	H	-	-	-	-	-	-	-	-	-	-	-
67	11	not last 4 weeks	H	-	-	-	-	-	-	-	-	-	-	-
68	7	not last 4 weeks	H	-	-	-	-	-	-	-	-	-	-	-
**#**	**Age (in years)**	**Vac. status**	**Group**	**Snap® Parvo (POCT-A)**	**Fassisi® Parvo (POCT-B)**	**Primagnost® Parvo H + K (POCT-C)**	**FASTest® PARVO Card (POCT-D)**	**Vetexpert Rapid Test CPV Ag® (POCT-E)**	**Anigen Rapid CPV Ag Test Kit® (POCT-F)**	**ImmunoRun® Parvovirus Antigen Detection Kit (POCT-G)**	**WITNESS® Parvo (POCT-H)**	**qPCR**	**Virus load/g faeces**	**Virus culture**
69	7	not last 4 weeks	H	-	-	-	-	-	-	-	-	-	-	-
70	3	not last 4 weeks	H	-	-	-	-	-	-	-	-	-	-	-
71	4	not last 4 weeks	H	-	-	-	-	-	-	-	-	-	-	-
72	2	not last 4 weeks	H	-	-	-	-	-	-	-	-	+	3,74E+06	-
73	6	not last 4 weeks	H	-	-	-	-	-	-	-	-	+	2,97E+06	-
74	2	not last 4 weeks	H	-	-	-	-	-	-	-	-	+	2,08E+06	-
75	5	not last 4 weeks	H	-	-	-	-	-	-	-	-	-	-	-
76	2	not last 4 weeks	H	-	-	-	-	-	-	-	-	-	-	-
77	10	not last 4 weeks	H	-	-	-	-	-	-	-	-	-	-	-

#	Age (in years)	Vac. status	Group	Snap® Parvo (POCT-A)	Fassisi® Parvo (POCT-B)	Primagnost® Parvo H + K (POCT-C)	FASTest® PARVO Card (POCT-D)	Vetexpert Rapid Test CPV Ag® (POCT-E)	Anigen Rapid CPV Ag Test Kit® (POCT-F)	ImmunoRun® Parvovirus Antigen Detection Kit (POCT-G)	WITNESS® Parvo (POCT-H)	qPCR	Virus load/g faeces	Virus culture
78	9	not last 4 weeks	H	-	-	-	-	-	-	-	-	-	-	-
79	4	not last 4 weeks	H	-	-	-	-	-	-	-	-	-	-	-
80	13	not last 4 weeks	H	-	-	-	-	-	-	-	-	-	-	-
81	3	not last 4 weeks	H	-	-	-	-	-	-	-	-	-	-	-
82	6	not last 4 weeks	H	-	-	-	-	-	-	-	-	-	-	-
83	11	not last 4 weeks	H	-	-	-	-	-	-	-	-	-	-	-
84	5	not last 4 weeks	H	-	-	-	-	-	-	-	-	-	-	-
85	10	not last 4 weeks	H	-	-	-	-	-	-	-	-	-	-	-
86	2	not last 4 weeks	H	-	-	-	-	-	-	-	-	-	-	-
87	6	not last 4 weeks	H	-	-	-	-	-	-	-	-	+	1,70E+06	-
88	4	not last 4 weeks	H	-	-	-	-	-	-	-	-	-	-	-
89	9	not last 4 weeks	H	-	-	-	-	-	-	-	-	+	2,60E+06	-
90	6	not last 4 weeks	H	-	-	-	-	-	-	-	-	+	2,83E+06	-
91	1	not last 4 weeks	H	-	-	-	-	-	-	-	-	-	-	-
92	2	not last 4 weeks	H	-	-	-	-	-	-	-	-	-	-	-
93	6	not last 4 weeks	H	-	-	-	-	-	-	-	-	-	-	-
94	2	not last 4 weeks	H	-	-	-	-	-	-	-	-	-	-	-
95	6	not last 4 weeks	H	-	-	-	-	-	-	-	-	-	-	-
96	8	not last 4 weeks	H	-	-	-	-	-	-	-	-	+	1,17E+06	-
97	9	not last 4 weeks	H	-	-	-	-	-	-	-	-	+	1,32E+06	-
98	6	not last 4 weeks	H	-	-	-	-	-	-	-	-	+	4,05E+05	-
99	4	not last 4 weeks	H	-	-	-	-	-	-	-	-	-	-	-
100	2	not last 4 weeks	H	-	-	-	-	-	-	-	-	+	1,64E+06	-
101	0.30	8 weeks 12 weeks	P	-	-	-	-	-	-	-	-	+	3,38E+10	-
102	0.19	not vac.	P	-	-	-	-	-	-	-	-	+	8,86E+11	-
103	0.25	unknown	P	-	-	-	-	-	-	-	-	+	4,00E+13	-
104	0.92	6 weeks 10 weeks	P	-	-	-	-	-	-	-	-	+	2,62E+12	-

105	0.25	6 weeks 8 weeks	P	-	-	-	-	-	-	-	-	+	1,32E+14	-
106	0.25	8 weeks	P	-	-	-	-	-	-	-	-	+	2,71E+10	-
107	0.83	un-known	P	-	-	-	-	-	-	-	-	+	1,44E+13	-
108	0.33	not vac.	P	-	-	-	-	-	-	-	-	+	9,40E+07	-
109	0.19	not vac.	P	+	+	+	+	+	+	+	+	+	1,00E+14	+
110	0.58	7 weeks 11 weeks	P	-	-	-	-	-	-	-	-	+	1,45E+09	-

#	Age (in years)	Vac. sta-tus	Grou p	Snap® Parvo (POCT-A)	Fassisi® Parvo (POCT-B)	Primag-nost® Parvo H + K (POCT-C)	FASTest® PARVO Card (POCT-D)	Vetexpert Rapid Test CPV Ag® (POCT-E)	Anigen Rapid CPV Ag Test Kit® (POCT-F)	ImmunoRun® Par-vovirus Antigen Detection Kit (POCT-G)	WIT-NESS® Parvo (POCT-H)	qPCR	Virus load/g faeces	Virus culture
111	0.25	un-known	P	-	-	-	-	-	+	-	+	+	1,41E+13	-
112	0.33	12 weeks	P	+	+	+	+	+	+	+	+	+	1,21E+14	+/-
113	0.42	8 weeks 12 weeks	P	-	-	-	-	-	-	-	-	+	2,64E+10	-
114	0.17	un-known	P	-	-	-	-	-	-	-	-	+	7,23E+07	-
115	0.25	un-known	P	+	-	+	+	+	+	+	+	+	3,32E+12	+
116	0.92	10 weeks 12 weeks	P	-	-	-	-	-	-	-	-	+	8,53E+09	-
117	0.58	un-known	P	-	-	-	-	-	-	-	-	+	3,40E+10	-
118	0.67	8 weeks 12 weeks	P	-	-	-	-	-	-	-	-	+	1,68E+06	-
119	0.21	not vac	P	-	-	-	-	-	-	-	-	+	1,47E+12	-
120	0.67	not vac	P	-	-	-	-	-	-	-	-	+	3,20E+07	-
121	0.42	not vac	P	+	-	-	-	+	+	+	-	+	1,45E+12	-
122	0.15	6 weeks	P	+	+	+	+	+	+	+	+	+	9,88E+14	-
123	0.19	un-known	P	-	-	-	-	+	-	-	-	+	1,48E+09	-
124	0.19	un-known	P	-	-	-	-	-	-	-	-	+	1,50E+12	-

#	Age (in years)	Vac. Sta-tus	Grou p	Snap® Parvo (POCT-A)	Fassisi® Parvo (POCT-B)	Primag-nost® Parvo H + K (POCT-C)	FASTest® PARVO Card (POCT-D)	Vetexpert Rapid Test CPV Ag® (POCT-E)	Anigen Rapid CPV Ag Test Kit® (POCT-F)	ImmunoRun® Par-vovirus Antigen Detection Kit (POCT-G)	WIT-NESS® Parvo (POCT-H)	qPCR	Virus load/g faeces	Virus culture
125	0.27	8 weeks	P	-	-	-	-	-	-	-	-	+	8,73E+12	-
126	0.33	un-known	P	+	+	+	+	+	+	+	+	+	2,85E+12	+
127	0.15	4 weeks	P	+	+	+	+	+	+	+	+	+	2,94E+13	+
128	0.08	not vac.	P	+	+	+	+	+	+	+	+	+	7,27E+13	+
129	0.10	4 weeks	P	+	+	+	+	+	+	+	+	+	5,43E+13	+
130	2.00	not vac.	P	+	+	+	+	+	+	+	+	+	1,76E+12	+
131	0.50	6 weeks 9 weeks	P	+	+	+	+	+	+	+	+	+	7,45E+11	+
132	0.25	un-known	P	+	+	-	-	+	+	+	+	+	2,04E+13	+
133	0.42	12 weeks	P	+	+	+	+	+	+	+	+	+	3,99E+12	+
134	0.67	6 weeks	P	+	+	+	+	+	+	+	+	+	1,36E+12	+
135	0.67	un-known	P	+	+	+	+	+	+	+	+	+	1,80E+11	+
136	1.67	un-known	P	+	+	+	+	+	+	+	+	+	2,58E+12	+
137	0.27	8 weeks	P	-	+	-	-	+	-	-	-	+	6,65E+09	-
138	0.19	un-known	P	-	-	-	-	-	-	-	-	+	3,85E+11	-

#	Age (in years)	Vac. status	Group	Snap® Parvo (POCT-A)	Fassisi® Parvo (POCT-B)	Primagnost® Parvo H + K (POCT-C)	FASTest® PARVO Card (POCT-D)	Vetexpert Rapid Test CPV Ag® (POCT-E)	Anigen Rapid CPV Ag Test Kit® (POCT-F)	ImmunoRun® Parvovirus Antigen Detection Kit (POCT-G)	WITNESS® Parvo (POCT-H)	qPCR	Virus load/g faeces	Virus culture
139	0.58	8 weeks 12 weeks	P	+	+	+	+	+	+	+	+	+	3,43E+14	+
140	0.15	unknown	P	+	+	+	+	+	+	+	+	+	4,21E+14	+
141	0.33	unknown	P	-	-	-	-	-	-	-	-	+	1,16E+10	-
142	0.10	unknown	P	-	-	-	-	-	-	-	-	+	5,00E+11	-
143	0.10	unknown	P	-	-	-	-	-	-	-	-	+	5,22E+10	-
144	0.10	unknown	P	+	+	-	-	+	+	+	+	+	1,25E+14	-
145	0.15	unknown	P	+	-	-	-	+	+	-	+	+	5,48E+13	+
146	0.17	unknown	P	+	-	-	-	+	+	+	-	+	3,96E+13	-
147	0.27	not vac.	P	+	-	+	-	+	+	+	+	+	8,00E+12	-
148	0.15	6 weeks	P	-	-	-	-	-	-	-	-	+	1,90E+11	+
149	0.15	unknown	P	-	-	-	-	-	-	-	-	+	1,55E+11	-
150	0.58	unknown	P	-	-	-	-	-	-	-	-	-	-	-

qPCR, real-time polymerase chain reaction, POCT, point-of-care test, Vac., vaccinated.
